# Effectiveness of an Evidence-Based Practice educational intervention with second-year nursing students^1^

**DOI:** 10.1590/1518-8345.2502.3026

**Published:** 2018-08-09

**Authors:** Desirée Mena-Tudela, Víctor Manuel González-Chordá, Agueda Cervera-Gasch, María Loreto Maciá-Soler, María Isabel Orts-Cortés

**Affiliations:** 2 PhD, Assitant Professor, Facultad de Ciencias de la Salud, Universitat Jaume I, Castellón de la Plana, Comunidad Valenciana, Spain.; 3 PhD, Professor, Facultad Ciencias de la Salud, Universidad de Alicante, San Vicente del Raspeig, Alicante, Spain.

**Keywords:** Nursing, Nursing Education, Education, Nursing, Baccalaureate, Evidence-Based Practice, Nursing Education Research, Nursing Research

## Abstract

**Objectives:**

to evaluate the effectiveness of an educational intervention on the knowledge, skills and attitudes of evidence-based practice among second-year nursing students.

**Method:**

a quasi-experimental before-and-after study. The study population consisted of 120 students enrolled in the Nursing Care in Healthcare Processes course. The educational intervention was based on theoretical and practical classes about the evidence-based practice process and the use of the critical incident technique during the clinical clerkship. Effectiveness was measured with the Evidence-Based Practice Competence Questionnaire in three paired measures using repeated-measures analysis of variance.

**Results:**

the mean scores of the Evidence-Based Practice Competence Questionnaire were 79.83 (CI 95% 78.63-81.03) for the basal measurement, 84.53 (CI 95% 83.23-85.83) for the intermediate measurement, and 84.91 (CI 95% 83.26-86.55) for the final measurement, with a statistically significant difference among the three paired measurements (p<0.001). There were statistically significant differences in Attitudes (p = 0.034) and Knowledge (p <0.001) but not in Skills (p = 0.137).

**Conclusion:**

this educational intervention based on theoretical and practical classes about the evidence-based practice process and the use of the critical incident technique during the clinical clerkship enhances evidence-based practice competence among second-year nursing degree students.

## Introduction

Evidence-based medicine (EBM) first appeared as a clinical learning strategy at McMaster Medical School and, because healthcare professionals from many fields currently adopt this approach in their clinical practice, EBM has evolved into evidence-based practice (EBP), a wider and more comprehensive concept^1^.

EBP involves the integration and implementation of the best available evidence, including clinical expertise and patients’ values and circumstances, in clinical decision-making^2^ ^-^ ^3^. Traditional EBP is a five-step process: Ask, Acquire, Appraise, Apply and Assess^3^. Recently, however, some researchers^4^ have developed a seven-step EBP process, with the addition of ‘Cultivation of a spirit of inquiry’ as a step zero and ‘Dissemination of the results’ as a sixth step. Both processes comprise other sources of evidence in addition to research evidence so that EBP has come to utilise more than research and now embraces interprofessional teams, patients and the best available evidence to optimise patient outcomes.

Despite the evidence that EBP improves patient outcomes and performance, incorporating EBP into clinical nursing remains a challenge^2^ ^-^ ^4^. This incorporation of EBP requires positive attitudes, knowledge and skills related to research. Clinical nurses highlight the lack of time, organisational support and abilities to search, critique and synthesise the literature^5^ ^)^ as barriers to the incorporation of EBP, probably because they were not educated on the EBP paradigm^6^. Today, the introduction of EBP education in nursing curricula is strongly recommended in the United States^7^, Australia^8^ and Europe^9^, and in some countries, such as the United Kingdom, EBP competence is compulsory in nursing degree programmes^10^. In Spain, EBP competence is not considered by the specific legislation that stipulates the minimum requirements for the verification of nursing degrees^11^, although some universities do make an effort to incorporate EBP education into their curricula^6^ ^,^ ^12^.

There are several books on teaching EBP that can help teachers and educators implement EBP education into their curricula^3^ ^-^ ^4^, and The Sicily Statement on EBP recommends the minimum standard educational requirements for training health professionals in EBP^1^. Moreover, the EBP education literature is extensive, and several systematic reviews have been conducted to evaluate the effectiveness of EBP training^12^ ^-^ ^13^. These studies assessing the effectiveness of EBP training focus mainly on short-term interventions^6^, although there are authors^14^who outline multifaceted interventions, such as combining lectures, computer lab sessions, journal clubs or portfolios as better ways to improve students’ knowledge, skills and attitudes as opposed to single interventions. Conversely, the American Association of Colleges of Nursing^7^ does not recommend the inclusion of all the stages of EBP in the baccalaureate nursing curricula as standard practice due to a lack of cognitive maturity among students^15^. The drawback is that the literature does not provide conclusive evidence on the best practices for EBP education, yet some relevant recommendations and areas for debate can be drawn. Furthermore, there is no consensus on the most appropriate year to start EBP training, although it seems that an early introduction to the EBP process increases students’ interest and skills^16^. As such, several authors support the idea that EBP education must be embedded as a cross-curricular competence^4^ ^,^ ^16^ ^-^ ^17^. Despite these conflicting theories, developing nursing students’ critical thinking skills is a requisite for teaching EBP^13^, which coincides with step zero (‘Cultivation of a spirit of inquiry’)^4^. This could be the most difficult step, but some studies show that problem-based learning^18^, journal clubs^19^ or critical incidents^20^ are learning strategies that promote critical thinking and EBP. While the development of critical thinking is a necessary step, information literacy is considered the most important skill at the bachelor’s level. Some authors^1^ ^,^ ^13^ offer recommendations on educational interventions during this step (theoretical instruction and supervised practical sessions with online content). Another recommendation is the need to integrate the learning of EBP as a routine in the clinical context and to provide continuous opportunities to apply EBP skills^1^ so that students can link EBP knowledge and skills with real clinical situations. To this end, some researchers^18^ ^,^ ^20^ ^-^ ^21^ have developed specific models and strategies to integrate the teaching of EBP with its practice.

**Figure 1:**
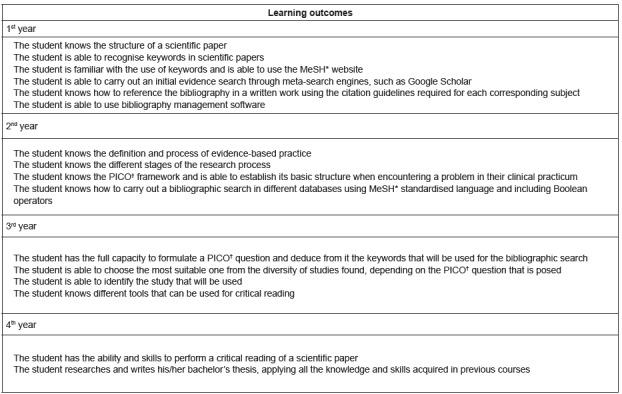
Learning outcomes of the cross-curricular evidence-based practice programme in the bachelor’s degree programme at the Universitat Jaume I, Castellón, Spain, 2017

The nursing degree was introduced at the Universitat Jaume I (Spain) in 2011 after the establishment of the European Higher Education Area^12^. The learning methodology of the nursing programme integrates theory, simulated practice and clinical practice through learning outcomes, so that students acquire knowledge in the classroom, skills in simulation rooms and opportunities to demonstrate their acquired competences in a clinical clerkship. During this clerkship, students are supervised by nurses who have passed an initial 40-hour training course covering topics related to the curriculum, the assessment of competences in a clinical clerkship and EBP^22^. The curriculum has four courses; 50% of them are based on clinical clerkship, and the teaching-learning process has at its core the minimum competences established by Spanish legislation^11^. Moreover, the curriculum incorporates a cross-curricular competence in EBP defined as the “capacity to assert clinical judgements to ensure that quality standards are met and that the practice is based on evidence”^12^. With the aim of accomplishing the progressive acquisition of this EBP competence, a four-year cross-curricular EBP programme was designed, based on recent literature and the main recommendations for EBP education. This programme includes learning outcomes and training activities for each year, as well as cross-curricular activities (Figure 1 shows an overview of the learning outcomes of the cross-curricular EBP programme). 

* Medical Subject Headings; † Population-Intervention-Comparison-Outcome.

To assess EBP education, some authors^23^ developed the Classification Rubric for EBP Assessment Tools in Education (CREATE) for classifying EBP learning assessment tools. They recommend the use of the Fresno test and the Berlin Questionnaire as validated tools, although they also highlight the need for further development and validation of EBP learning assessment tools; these tools have not been validated in Spanish. Thus, the Evidence-Based Practice Competence Questionnaire (EBP-COQ) has recently been developed and validated to evaluate the self-perceived level of EBP competence in Spanish nursing students^24^. The EBP-COQ consists of 25 items organised in three dimensions (Skills, Knowledge and Attitudes), with 55.55% of the variance explained. The items are measured on a 5-point Likert scale (1. Strongly disagree, 2. Disagree, 3. Neither agree nor disagree, 4. Agree, 5. Strongly Agree). The questionnaire has nine items scored in reverse order. The Cronbach’s alpha for the overall questionnaire was 0.888 (Attitude α = 0.94, Skills α = 0.756; Knowledge α = 0.8)^25^.

Therefore, the main objective of this study was to evaluate the effectiveness of the educational intervention implemented in the second year of the nursing degree at Universitat Jaume I (Spain) on students’ knowledge, skills and attitudes towards EBP.

## Methods

A quasi-experimental before-and-after study was conducted among a group of second-year nursing students enrolled in the nursing degree programme at Universitat Jaume I (Spain).

The study population consisted of 120 students enrolled in the Nursing Care in Healthcare Processes course during the second year of the nursing degree programme at Universitat Jaume I during the 2013-2014 and 2014-2015 academic years. This course is taken during the second semester of the second year and is composed of a period of theory and clinical simulation and a period of clinical clerkship in adult inpatient units.

The sample size was calculated with the GRANMO software package. A minimum sample size of 65 students was required to detect a difference of 0.2 units in the EBP-COQ score, with a standard deviation of 0.4^25^, considering an alpha risk of 0.05 and a beta risk of 0.05 in a one-sided means test in a paired group, taken from a study of the EBP-COQ among Spanish nursing students^25^. A drop-out rate of 20% was considered. Considering this, all students enrolled in the Nursing Care in Healthcare Processes course in the academic years of 2013-2014 and 2014-2015 were included. 

A student’s failure to give consent to participate was considered an exclusion criterion. Furthermore, students who did not complete the clinical clerkship, who abandoned their studies during the clerkship, or who took the course during special periods (added time periods in which students can make up the hours needed to complete their clinical clerkship) were excluded. Students who did not complete the three measures were also excluded from the study.

The educational intervention was carried out with second-year nursing degree students and was framed within the cross-curricular EBP programme described in Figure 1. It is important to consider previous knowledge gained in the first year and the continuity of the programme in subsequent years.

This educational intervention consists of two hours of EBP theory and two hours of computer lab sessions. In the first session, terms related to EBP are defined, and students are encouraged to reflect on the material and to employ critical thinking in the use of research tools. The second session is a practical exercise on information literacy. 

During the clinical clerkships (12 weeks), the critical incident technique is used to stimulate the implementation of the acquired knowledge and skills. Students identify a minimum of eight critical incidents related to the content of the course and their daily practice and document the critical incident information (case description, emotions, coping with the case, result of action, dilemmas, learning). Furthermore, students develop a clinical question (following the Population-Intervention-Comparison-Outcomes format) for each critical incident and try to resolve it through a literature search. One lecturer is responsible for providing support and feedback to a group of 8 to 10 students. Students receive a lecture explaining the critical incident technique and its methods before starting the clinical clerkship. The study was conducted between September 2013 and June 2015.

The effect of the educational intervention on the students’ knowledge, skills and attitudes towards EBP was measured with the EBP-COQ^26^. A baseline measurement with the EBP-COQ was performed at the beginning of the semester in both academic years, and socio-demographic variables (age and sex) were also collected. An intermediate measurement was then performed two months later, before the start of clinical clerkship, and a final measurement was recorded at the end of the 12-week clinical clerkship period. The three measurements were carried out during classes at the university.

A descriptive analysis of the categorical variables (frequency distributions, proportions and 95% confidence intervals [CI 95%]) and continuous variables (mean, median, standard deviation [SD], minimum and maximum and CI 95%) was performed.

To compare the categorical variables, a Z-proportions comparison test was applied using the Bonferroni correction. Repeated-measures ANOVA with the appropriate post hoc contrast was used to compare the three measures of the EBP-COQ, and if normality of the variables was not met, the Friedman test was used. A p-value <0.05 for bilateral significance was assumed, and the statistical analysis was performed using SPSS software for Windows v20.

This study was approved as an educational innovation project (Grant no.: 045/14) by the Nursing Department Council of the Universitat Jaume I, and permission to use the EBP-COQ was obtained from its authors. Students were invited to take part in the study by means of a cover letter, which included the introduction, objectives, and methodology of the study and a request for their consent. The questionnaires were completed on a voluntary basis and the anonymity of the students was maintained throughout the entire process using a previously described procedure in compliance with Spanish legislation on personal data protection. The ethical principles of the Helsinki Declaration were followed.

## Results

The response rate was 69.17% (n=83). Two students abandoned their studies during the clinical clerkship, 17 students conducted their clinical clerkship in the special periods, 8 students did not complete their clinical clerkship and 10 students did not respond to the three measurements. Of the participants, 84.3% (n=70) were women, with no significant differences between courses (p= 0.203). The average age was 21.6 years (SD ± 5.62), with a minimum of 19 and a maximum of 48, with no significant differences between courses (p = 0.194; 95% CI 20.37-22.83 years).

The mean score of the EBP-COQ for the overall sample was 79.83 points (SD ± 4.86, 95% CI 78.63-81.03) for the baseline measurement, 84.53 points (SD ± 5.29, 95%, CI 83.23-85.83) for the intermediate measurement and 84.91 points (SD ± 7.28, 95% CI 83.26-86.55) for the final measurement. There were significant differences among the three measurements (p < 0.001), between the baseline and the intermediate measurements (p < 0.001) and between the baseline and the final measurements (p<0.001) but not between the intermediate and the final measurements (p=0.092). Table 1 shows the descriptive analysis of the three dimensions (Attitudes, Knowledge, and Skills). 

**Table 1:** Descriptive analysis of the dimensions of the EBP-COQ*. Universitat Jaume I, Castellón, Spain, 2017

		BM^†^	IM^‡^	FM^§^
Attitudes	Mean	3.47	3.55	3.55
	SD^||^	0.28	0.32	0.39
	95% CI^¶^	3.42-3.56	3.51-3.66	3.49-3.68
Knowledge	Mean	2.82	3.36	3.45
	SD^||^	0.41	0.35	0.41
	95% CI^¶^	2.70-2.91	3.29-3.46	3.41-3.50
Skills	Mean	2.94	2.92	3.02
	SD^||^	0.34	0.29	0.35
	95% CI^¶^	2.85-3.00	2.86-3.01	2.90-3.05

*EBP-COQ: Evidence-Based Practice Competence Questionnaire; †BM: Baseline Measurement; ‡IM: Intermediate Measurement; §FM: Final Measurement; ||SD: Standard Deviation; ¶95% confidence interval.

Table 2 shows the descriptive analysis of the items in the Attitudes category. Items 1, 3, 5 and 7 in this category obtained mean scores above 4 in all three measurements, and the ANOVA revealed a significant difference for all items in this category, except for items 1 (p=0.099), 10 (p=0.065) and 11 (p = 0.441). All items in the Skills category showed significant differences among the three measurements (Table 3).

**Table 2:** Descriptive analysis: Attitudes. Universitat Jaume I, Castellón, Spain, 2017

		Min*	Max^†^	Mean	SD^‡^	p^§^
1. Evidence-based practice helps in regard to making decisions in clinical practice	BM^||^	3	5	4.29	0.65	0.099
	IM^¶^	3	5	4.47	0.59	
	FM**	2	5	4.40	0.62	
2. I am confident that I will be able to critically evaluate the quality of a scientific article	BM^||^	2	5	3.67	0.81	<0.001
	IM^¶^	2	5	3.81	0.70	
	FM**	1	5	3.89	0.84	
3. The application of evidence-based practice will help to better define the nurse’s role	BM^||^	3	5	4.08	0.69	<0.001
	IM^¶^	2	5	4.23	0.72	
	FM**	2	5	4.22	0.74	
4. The nursing contract should include time to read scientific papers and make a critical appraisal of them	BM^||^	2	5	3.74	0.74	0.051
	IM^¶^	1	5	3.94	0.81	
	FM**	2	5	3.88	0.80	
5. Widespread evidence-based practice implementation will allow increased nursing autonomy from other professions	BM^||^	3	5	4.13	0.74	0.023
	IM^¶^	2	5	4.24	0.72	
	FM**	3	5	4.30	0.61	
6. When I work as a nurse, I will be pleased if evidence-based practices are implemented in my practice	BM^||^	2	5	3.97	0.63	0.018
	IM^¶^	1	5	4.16	0.83	
	FM**	1	5	4.12	0.86	
7. The application of evidence-based practice improves patients’ healthcare outcomes	BM^||^	3	5	4.21	0.66	0.055
	IM^¶^	3	5	4.39	0.62	
	FM**	2	5	4.39	0.69	
8. In the future, I wish to contribute to the application of evidence-based practice	BM^||^	1	5	3.80	0.82	0.047
	IM^¶^	1	5	3.83	0.96	
	FM**	1	5	3.75	1.05	
9. I do not like reading scientific articles^††^	BM^||^	1	5	2.12	0.86	0.015
	IM^¶^	1	5	1.95	0.82	
	FM**	1	5	2.08	0.92	
10. Patient care will undergo minor changes with the application of evidence-based practice^††^	BM^||^	1	4	2.04	0.74	0.065
	IM^¶^	1	4	1.87	0.82	
	FM**	1	5	1.86	0.89	
11. I am pleased that evidence-based practice is only a theoretical movement that is not implemented in practice^††^	BM^||^	1	5	1.83	0.82	0.441
	IM^¶^	1	4	1.67	0.68	
	FM**	1	4	1.76	0.82	
12. If I had the opportunity, I would attend a course on evidence-based practice	BM^||^	2	5	3.53	0.79	0.003
	IM^¶^	1	5	3.58	0.84	
	FM**	1	5	3.49	1.05	
13. I would like to have better access to published scientific evidence on nursing	BM^||^	1	5	3.83	0.89	0.017
	IM^¶^	1	5	4.13	0.67	
FM**	1	5	4.10	0.81		

*Min: minimum;†Max: maximum; ‡SD: Standard Deviation; §Friedman test; ||BM: Baseline Measurement; ¶IM: Intermediate Measurement; **FM: Final Measurement; ††Items written in reverse order.

**Table 3:** Descriptive analysis: Skills. Universitat Jaume I, Castellón, Spain, 2017

		Min*	Max^†^	Mean	SD^‡^	p^§^
1. I feel able to develop a clinical question to start researching the best scientific evidence	BM^||^	1	5	3.13	0.79	<0.001
	IM^¶^	2	5	3.82	0.60	
	FM**	2	5	4.10	0.74	
2. I do not feel able to search for scientific evidence in the principle health sciences databases^††^	BM^||^	1	5	2.71	1.01	<0.001
	IM^¶^	1	5	2.11	0.79	
	FM**	1	5	2.18	1.00	
3. I do not feel able to search for scientific information about the subject in the most important bibliographic indexes^††^	BM^||^	1	5	2.63	0.92	<0.001
	IM^¶^	1	5	2.18	0.81	
	FM**	1	4	2.16	0.87	
4. I feel able to critically evaluate the quality of a scientific article	BM^||^	1	5	2.89	0.83	<0.001
	IM^¶^	1	5	3.39	0.82	
	FM**	1	5	3.51	0.87	
5. I do not feel able to analyse whether the results obtained in a scientific study are valid^††^	BM^||^	2	5	3.08	0.78	<0.001
	IM^¶^	1	4	2.41	0.81	
	FM**	1	4	2.39	0.88	
6. I feel able to analyse the practical utility of a scientific study	BM^||^	2	4	3.20	0.77	<0.001
	IM^¶^	2	5	3.65	0.61	
FM**	2	5	3.78	0.73		

*Min: minimum; †Max: maximum; ‡SD: Standard Deviation; §Friedman test; ||BM: Baseline Measurement; ¶IM: Intermediate Measurement; **FM: Final Measurement; ††Items written in reverse order.

All items in the knowledge category showed significant differences among the three measurements (Table 4). Item 1 obtained an average baseline measurement of 2.43 (SD ± 0.88) points, increasing in the following measurements to above 4 points.

**Table 4:** Descriptive analysis: Knowledge. Universitat Jaume I, Castellón, Spain, 2017

		Min*	Max^†^	Mean	SD^‡^	p^§^
1. I know how to develop clinical questions organised in the PICO^||^ format	BM^¶^	1	5	2.43	0.88	<0.001
	IM**	2	5	4.16	0.75	
	FM^††^	1	5	4.36	0.69	
2. I know the principal sources that offer information that has been revised and catalogued from the evidence point of view	BM^¶^	1	5	2.49	0.92	<0.001
	IM**	1	5	3.94	0.86	
	FM^††^	2	5	4.29	0.80	
3. I do not know the most important characteristics of the principal investigation designs^‡‡^	BM^¶^	1	5	2.92	1.03	<0.001
	IM**	1	4	2.28	0.86	
	FM^††^	1	5	2.17	0.95	
4. I know the different levels of evidence of the investigation study designs	BM^¶^	1	5	2.61	0.96	<0.001
	IM**	1	5	3.59	0.88	
	FM^††^	1	5	3.74	0.92	
5. I do not know the different degrees of recommendation about adopting a certain procedure or health intervention^‡‡^	BM^¶^	1	5	3.39	0.86	<0.001
	IM**	1	4	2.53	0.73	
	FM^††^	1	5	2.40	0.92	
6. I know the principal measures of association and potential impact that allow me to evaluate the magnitude of the analysed effect in research studies	BM^¶^	1	5	3.08	1.07	<0.001
	IM**	1	5	3.69	0.99	
	FM^††^	1	5	3.83	0.94	

*Min: minimum; †Max: maximum; ‡SD: Standard Deviation; §Friedman test; ||Population-Intervention-Comparison-Outcome; ¶BM: Baseline Measurement; **IM: Intermediate Measurement; ††FM: Final Measurement; ‡‡Items written in reverse order.

## Discussion

The main goal of this study was to evaluate the effectiveness of an educational intervention carried out in the second-year nursing degree programme at Universitat Jaume I (Spain) on students’ knowledge, skills and attitudes towards evidence-based practice. This educational intervention was embedded in the cross-curricular evidence-based practice programme developed in the nursing degree programme at the Universitat Jaume I. It was based on recommendations from the literature regarding the use of theoretical and practical classes on the evidence-based practice process^1^ and the development of students’ critical thinking^12^ by using the critical incident technique^19^, and it was developed as a way to provide continuous opportunities to apply evidence-based practice skills during clinical clerkships^1^. Moreover, the effectiveness was measured using the EBP-COQ, a validated tool developed in Spanish to measure the knowledge, skills and attitudes of nursing students^24^ ^-^ ^26^.

Our results show that this educational intervention can improve students’ overall evidence-based practice competence, primarily in the Knowledge and Attitudes dimensions. However, there were no statistically significant differences in the Skills dimension, although the analysis of this dimension by items showed significant differences in all cases, which reinforces the educational intervention. The factors that may be involved include the influence of the clinical tutors, their relationship with the students and a possible lack of evidence-based practice knowledge and skills. Students perceive clinical tutors as role models, so involving tutors in the learning of evidence-based practice is crucial to improving students’ evidence-based practice competence^20^ by implementing journal clubs^18^ or by involving them in critical incident analysis. Moreover, students can promote evidence-based practice within clinical settings by forming partnerships with clinical nurses^18^. Another factor may be the difficulty students encounter in accessing electronic resources during their clinical clerkships. The use of mobile devices with Internet access may be a solution to this issue by providing fast access to evidence at the point of care; apps have recently been developed to effectively enhance students’ evidence-based practice skills^24^.

The first part of this educational intervention is conducted at the university, while the second occurs during the clinical clerkship. The comparison of means showed significant differences between the baseline and intermediate measurements but not between the intermediate and final measurements, thus suggesting that the first part of the educational intervention had a greater positive effect compared to the part carried out during the clinical clerkship. Other studies have evaluated the effectiveness of specific courses on research or evidence-based practice on students’ knowledge, skills and attitudes^6^ or on students’ cognitive load and learning performance^27^ with good results, but they have not addressed learning integrated within the clinical clerkships themselves. However, the improvements in students’ knowledge, attitudes and skills observed in evidence-based practice are greater when learning it is integrated into clinical clerkships^12^ ^,^ ^18^.

It is necessary to consider that this educational intervention was carried out among second-year nursing degree students and was framed within the cross-curricular evidence-based practice programme described earlier. Thus, it is important to consider that students in our programme had learned statistics and epidemiology in their first year, as some authors^6^ recommend; moreover, they had the fundamentals of evidence-based practice knowledge and skills gained in the first year, with further continuity of the programme in subsequent years. There are references regarding the implementation of similar cross-curricular evidence-based practice programmes proposed in the bachelor’s degree programme in nursing at the Universitat Jaume I^14^ ^-^ ^28^, although results regarding the educational interventions implemented or the overall programme are lacking. In our case, this is a partial assessment of the cross-curricular evidence-based practice programme, and it will be necessary to assess the effectiveness of the educational interventions carried out in forthcoming academic courses, as well as the overall results of the programme. 

Some limitations of this study must be considered. No control group was established, and the sample could not be randomised in the assessment of the educational intervention. This is because it was part of the cross-curricular EBP programme, open to all students enrolled in the Nursing Care in Healthcare Processes course, and because the nursing degree at Universitat Jaume I (Spain) has only recently been implemented. In subsequent academic courses, there were new interventions related to evidence-based practice that had to be measured. Moreover, the study sample was limited to two academic years and was carried out in a single institution.

The EBP-COQ is a robust and validated instrument for measuring the effectiveness of educational interventions on evidence-based practice, although it is based on students’ perceptions rather than on objective data. The use of subjective measures is suitable, however, because of the correlation between self-reported and objective assessments of evidence-based practice competence^6^.

## Conclusion

The findings of this study show that an educational intervention based on theoretical and practical classes on the EBP process and the use of the critical incident technique during clinical clerkship enhances EBP competence among second-year nursing degree students. However, the effect of the educational intervention is lower during clinical clerkships; factors related to clinical tutors, the use of technologies or the use of research may affect these results. Nevertheless, the findings of this study may be of interest to other universities since the existing literature does not provide sufficient evidence regarding the ideal model or the most appropriate training in EBP.
